# Integration of Environmental Data Into Electronic Health Records for Clinical and Public Health Decision Making: A Viewpoint on Expanding Development in the United States

**DOI:** 10.2196/76396

**Published:** 2025-08-29

**Authors:** Zade Akras, Caleb Dresser, Henry Ashworth

**Affiliations:** 1 Harvard Medical School, Boston, MA, United States; 2 Hikma Health, San Jose, CA, United States; 3 Beth Israel Deaconess Medical Center, Boston, MA, United States; 4 Department of Emergency Medicine, Highland Hospital, 1411 E 31st St, Oakland, CA, 94610, United States, 1 8052152433

**Keywords:** digital health, electronic medical records, environmental health, climate change, social determinants of health

## Abstract

Electronic health records are often extracted and combined with environmental data to conduct research or public health surveillance. However, to date, electronic health record systems do not integrate environmental data to aid real-time decision-making that could mitigate the health impacts of environmental hazards, including those related to climate change. Pursuing this goal requires enhancements to health record systems and modifications to the financial incentives driving health care innovation and delivery.

## Introduction

The impact of climate change on human health is projected to be substantial. The World Health Organization estimates that climate change will lead to an additional 250,000 deaths per year, with a direct annual cost of US $2 to $4 billion by 2030 [1]. The health effects of climate change are mediated through changes in environmental factors including temperature, precipitation, air quality, water quality, and weather extremes and variability [2]. A review of 94 systematic reviews and the 2023 report of the *Lancet Countdown* identified a wide variety of detrimental associations between climate change, climate-responsive hazards and environmental conditions, and health outcomes [3,4]. These relationships are diverse, ranging from heat-attributable mortality to impacts on food security and infectious disease dynamics [3,4].

According to the World Health Organization, 3.6 billion people are “highly susceptible” to the impacts of climate change, mostly in low-income countries with health systems inadequately prepared for acute or chronic environmental changes [1]. Developing “climate-resilient” health systems, which involves restructuring health care policy and delivery, can help address the health impacts of climate change [5].

**Figure 1.**
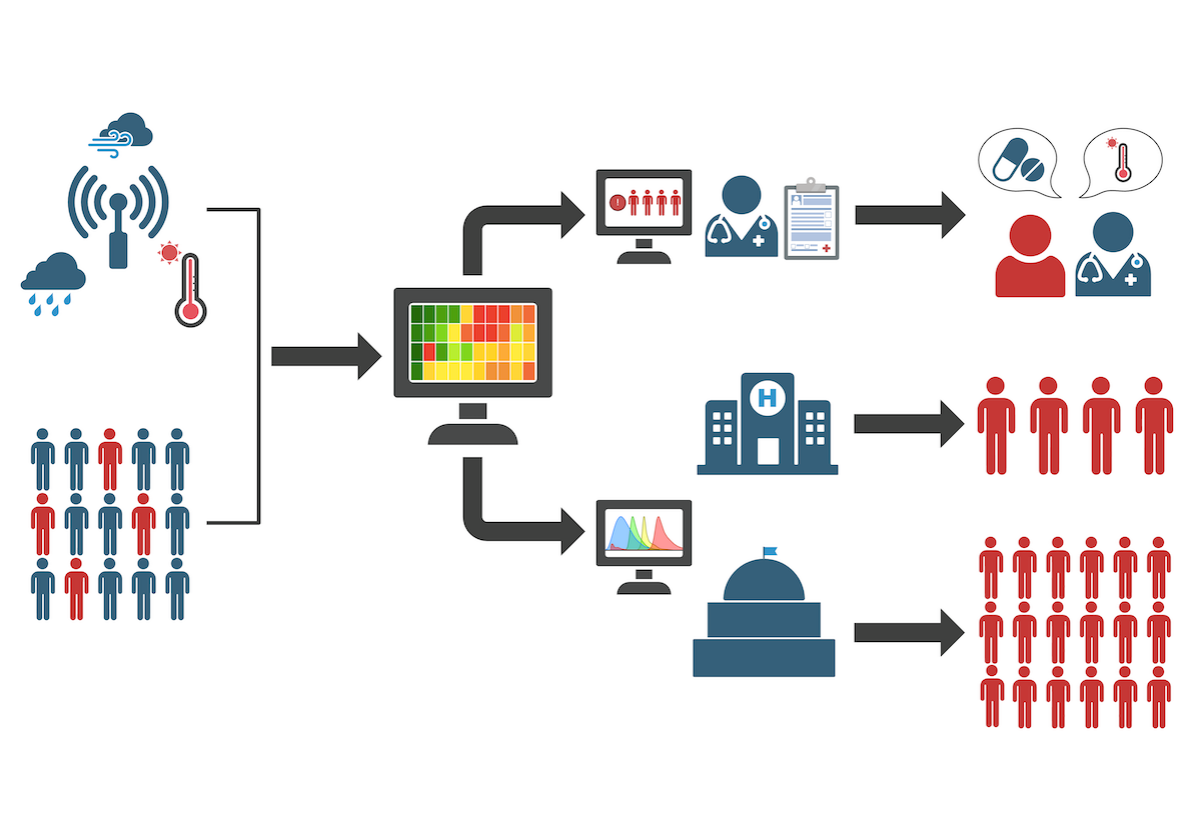
Workflow of an integrated health and environmental record system (EHR). An integrated EHR (Identified by the computer system) would capture environmental data from local and remote sensing technologies (indicated on the left by the sensor measuring climate). These data are integrated with health record information to identify at-risk patients (red figures). This system would produce three major outputs. At the individual level (top flow), these data can guide targeted interventions to mitigate disease exacerbations through physician actions, including patient counseling and medication prescriptions. At the health system level (middle flow) and government level (bottom flow), these data can guide forecasting and policymaking for population-level health to affect public health interventions, to target at-risk patients on a larger scale [9].

Digital tools such as electronic health records (EHRs) present an opportunity to improve health outcomes through patient risk stratification, decision support tools, and public health monitoring (Figure 1) [6]. However, regarding environmental exposures and climate-responsive hazards, these efforts have mostly focused on using EHR claims data for posthoc research and monitoring, and they have not been robustly applied for real-time risk stratification or decision support [7,8].

In this viewpoint, we describe existing environmental and digital health infrastructure, environmental data sources that can be used in various settings, obstacles to more widespread environment-informed digital health interventions, and opportunities for improved integration of environmental data with EHRs.

## Current Medical Record and Environmental Data Innovations

### Post-Hoc Electronic Health Record Research

In the past decade, there has been an increasing effort to align social determinants of health (SDoH) and environmental data with EHR datasets for research purposes. These studies have typically used EHR claims data or deidentified extracted datasets [7]. A systematic review of studies that spatially analyzed EHR claims found a total of 128 articles; however, only 11 of these articles included outdoor air quality or climate, and none incorporated real-time environmental data into EHRs for clinical decision-making. All 11 environment-related articles extracted data from EHRs and compared it with environmental data sources to analyze the health impacts of environmental factors, such as air quality, temperature, and pollution on maternal-fetal conditions or asthma [7]. A separate systematic review of extracted EHR data for epidemiological research similarly identified six studies that linked health outcomes with exposures in the physical environment, such as air pollution or green spaces. However, none of these studies used live data within EHR systems to proactively address these exacerbations [8]. An editorial has called for integrating sensor-derived data from smart home systems into EHRs, but these systems are still conceptual [10].

Some studies have used nonenvironmental SDoH variables such as home address or food insecurity as proxies for overall risk related to location or general environment [8,10-12]. Aggregation of these SDoH variables into “community vital signs” offers a rapid assessment that, if integrated directly into EHRs, would enable clinicians to rapidly screen individuals most susceptible to social or environmental impacts on health [11]. Three case studies have previously demonstrated the feasibility of integrating SDoH metrics into EHRs; these integrations enabled screening and referral to community agencies for food insecurity, generation of utility shut-off protection letters for patients with chronic illness, and referrals to Veterans Affairs homelessness programs [12]. Barriers to widespread adoption include lack of incentives, training, and privacy. While these studies report the collection and analysis of social data, they did not include specific environmental data [10-12].

### Public Health Surveillance

**Figure 2.**
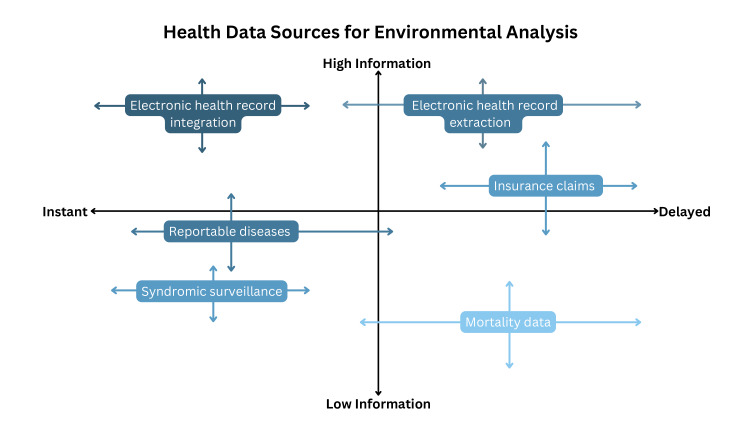
Health data sources for environmental analysis. We outline the approximate utility of different health data sources based on the richness of the information they offer (in terms of time, space, and data detail) as well as the timelines which the data is generated and can be acted upon. There is inherent variation to each of these data sources based upon how they are collected and used across hospital and public health systems.

Efforts have been made to integrate the analysis of health data with environmental data to improve public health surveillance and monitoring in the United States [13,14], Japan [15], Europe [16], and Canada [17]. In the United States, the Centers for Disease Control created the National Environmental Public Health Tracking Network (Tracking Network), which integrates data from state and local health departments into an outcomes tracking network [13]. Research has shown that this network directly improved 35 public health interventions, mostly through enhanced surveillance [16]. However, the Tracking Network does not provide publicly available real-time data to facilitate rapid response, does not report community- or individual-level data below the county level, and has focused on specific health outcomes [13,14]. The Tracking Network and similar systems are optimized to protect patient privacy and provide large-scale public health tracking, syndromic surveillance, and, in some cases, risk projections (eg, HeatRisk, which forecasts the risk of heat-related health incidents) [18]. However, individual health care institutions or municipalities responding to rapidly evolving environmental hazards may require higher-resolution, real-time local data to guide decisions. Additionally, countries that lack centralized health care record systems, such as the United States, face additional challenges in setting up comprehensive public health surveillance systems, given the fragmentation of patient data and data privacy concerns [1]. Health data sources suitable for analysis with environmental data are presented in Figure 2, along with information on their relative depth of information and the timeliness with which they can be used.

## Available Environmental Data

Environmental data sources useful for clinical decision-making and public health surveillance include national and local weather, climate, pollution, and toxin exposure data. Several currently available data sources in the United States can provide temperature, air quality, and water quality information, drawing on both in situ measurements and remote sensing.

Local environmental data can be accessed through the National Oceanic and Atmospheric Administration, which provides a free archive of weather and climate data, including temperature, precipitation, and wind [19]. However, this resource is limited by the existing distribution of weather stations, which are often found in locations removed from where most people live, such as airports. More expensive, local environmental monitoring can be achieved with meteorological, water, and pollution sensors [20]. Although this may increase the initial start-up cost for implementing an integrated EHR and environmental health system, it could drastically improve the accuracy of environmental data and bring resolution to the community level. Innovation in the design of remote sensors and modeling from sensor data is expected to continue driving down the cost of implementing these solutions [20].

Raw and processed data sources for temperature, air quality, and water quality measurements can also be collected using remote sensing technology, which uses sensors on aircraft or satellites to record emitted or reflected energy from the earth’s surface [21,22]. Global satellite-based observations are already proving valuable for assessing disaster situations [22,23]. Remote sensing allows a vast amount of information to be gleaned from the electromagnetic spectrum, providing detail not only on physical landscapes such as potential flood zones or cooling areas but also on water and air quality [22]. The Copernicus Land Monitoring Service and Earth Engine Data Catalog provide national and global data for temperature sources [23,24]. A significant benefit of the Copernicus data set is its application program interface that can be integrated into EHR programs and provide information updated hourly [24]. For air quality, the Esri ArcGIS Living Atlas of the World and OpenAQ are ArcGIS-powered data repositories with multiple free national and international data sets [25]. Although both options can provide real-time data and have good coverage for major cities, accurate data for rural areas may be unreliable [26]. For water quality, several remote assessment and monitoring options exist. In the United States, the US Geological Survey Water Data for the Nation collects various metrics of water quality from more than 430,000 sites [27]. Additionally, the National Aeronautics and Space Administration’s remote sensing observations can assess total suspended sediment levels, turbidity, and chlorophyll concentrations in inland lakes and other water sources [28].

While opportunities to source environmental data both locally and remotely are broad and are expanding rapidly through technological advancements, these modalities have limitations as surrogates for the microenvironments in which people live and spend most of their time. In situ sensors can improve the understanding of these locations, including indoor environments [10].

## Barriers to Innovation

Current EHR systems do not integrate real-time environmental data with clinical data [29-31]. There is an opportunity to enhance clinical and public health decision-making through such systems, but integrating environmental exposure information into EHR systems is challenging due to both structural barriers and lack of financial incentives.

The health data needed to implement this integration require the alignment of patient medical records with information on structural vulnerabilities and environmental exposures. Health and social data may be interspersed across multiple health systems and, consequently, across many EHRs with which a patient interacts [29,30]. Health systems may use their EHRs to construct public health registries independently, but these are limited to patients within the system and cannot capture longitudinal data from encounters with other systems [29,31]. The use of EHRs for public health surveillance is further limited by a lack of standardization across systems and the high resource demands of maintaining algorithms to extract relevant data [32]. Finally, information sharing between clinical and social service providers requires adequate data privacy and security considerations [12,32]. Given the sensitive and complex nature of the data being shared—including both protected health information and geospatial data, data exchange standards such as Fast Healthcare Interoperability Resources (FHIR) must be followed. Maintaining FHIR will be particularly difficult across multiple health systems given the integration of data from different EHR systems and ensuring that patients consent to exchange between each EHR and with other organizations such as public health authorities. Collectively, these barriers make it challenging to create a comprehensive, high-quality record of a patient’s medical conditions, social circumstances, and environmental exposures.

Problematic financial incentives exacerbate the logistical challenges of data retrieval and sharing. Hospitals may view patient data as an asset and be reluctant to share it with competitors [33]. EHR vendors seeking to capitalize on data transfers may charge high fees for this functionality or, when they are dominant in a given market, may obstruct cross-vendor exchange to encourage adoption of their system [30,31]. When comprehensive patient profiles do exist, they are often commodified and not readily available to patients, physicians, or other public health agencies [34].

Progress is being made in addressing these challenges. One solution to the fragmentation of EHRs is the creation of registries by public health agencies that encompass health systems within their jurisdiction. The New York City Department of Health and Mental Hygiene created a Population Health Registry, which directly interfaced with the EHRs of New York City clinics and tracked conditions managed by primary care practices in all five boroughs [35]. The Centers for Medicaid and Medicare Services (CMS) and the Administration for Strategic Preparedness and Response developed emPOWER, a database of Medicare beneficiaries requiring essential health services, to inform local officials during public health emergencies [36]. However, access to this dataset is limited by privacy restrictions, which reduce its resolution and usefulness outside of the limited use cases for which it was created [35,36].

One of the main barriers to creating an integrated health and environmental record is the lack of financial incentives for both public and private payers. Currently, the US federal government, through CMS, considers services addressing SDoH for reimbursement, but strictly environmental factors are not included [37]. Since it has been well-documented that environmental exposures and risks have a significant impact on health, adding these factors to reimbursement criteria could stimulate new innovation to address preventable environmentally-responsive diseases [38,39]. While considering social and environmental variables may burden hospitals with added complexity and cost, shifts toward value-based payment models could align financial incentives with the delivery of environmentally-informed preventative care [37,38]. Encouraging clinicians to document environmental impacts has also been proposed as a means to improve environmental health epidemiology by quantifying the health impacts and financial costs of environmental triggers [38].

However, the lack of reimbursement for environment-related codes limits their more widespread use [38,39], and challenges related to the attribution of individual cases to specific environmental variables need to be addressed. Private sector EHR innovation related to environmental data may be limited by the start-up costs and expertise needed to create an integrated health and environmental record system and the lack of clear financial incentives.

## Opportunities for Innovation

Climate and environmental data have been shown to improve health outcomes through proactive public health interventions [13-17]. There may be untapped potential in real-time environmental data integration to support clinical decision-making. We hypothesize that at the bedside, environmental data could support risk stratification tools to flag patients who are at high risk for complications of chronic disease from environmental stressors, helping clinicians provide targeted counseling, prescriptions, or connections to external resources. For example, if a patient with chronic obstructive pulmonary disorder (COPD) presents to a clinic and air quality is predicted to be poor in the near future, the EHR could prompt clinicians to provide counseling on lung protection during air pollution events and medication refills.

Incentivizing the development of health-environment systems can come from avenues in both the public and private sectors [38,39]. We believe that in the public sector, adding reimbursable billing codes for environment-related exposures, counseling, or testing would increase opportunities for reimbursement through CMS. In practice, adding environmental screening questions under the SDoH model could motivate fee-for-service health care providers to improve care for patients impacted by environmental exposures, and would provide a reimbursement mechanism to support this. From our perspective, private and capitated health systems would need to see the savings potential of pursuing innovation in this space. As each year new research is connecting additional relationships between environmental exposures and health outcomes ranging from oral cancers to infectious diseases, we suggest that a health and environmental database could help payers understand risk factors and intervention opportunities, including opportunities for net cost savings through enhanced prevention. Further research is needed to show both improvements in patient outcomes and the cost-effectiveness of combined health and environmental record systems.

## Use Case

Below is a use case for how this approach could be deployed in a moderately sized health system.

A rural town in the United States is experiencing increasingly severe heatwaves as climate change leads to hotter and drier weather. The town is served by a single public hospital and a small public health department. To help distribute their limited resources, the town’s health system implements an integrated environmental and health records system to help coordinate care for its aging population with multiple comorbidities.

During a summertime drought, a heat wave is predicted to hit the town with temperatures averaging 102 °F during the day. Using this information, the public health department quickly establishes cooling centers at accessible community locations, specifically those closest to elderly patients with lower socioeconomic status and who are unlikely to have access to air conditioning. Using the integrated EHR and environmental data system to prioritize their efforts, outreach teams make direct contact with vulnerable residents to ensure they are aware of the centers and have transportation to them. These patients are also flagged if they visit the emergency department or a primary care provider, allowing patient-specific counseling to be provided regarding medication management and access to cooling and other key services.

As the heat wave peaks, wildfires break out, releasing harmful smoke and increasing airborne pollutants such as PM2.5 and PM10. The environmentally integrated EHR system detects this increase, alerts the local hospital, and creates a list of residents with asthma and COPD who are most at risk given their specific comorbidities and demographics. Health care providers use these alerts to identify patients with respiratory diseases who need urgent care, medication refills, or preventive advice, and to prioritize telehealth services, enabling vulnerable residents to consult remotely and avoid exposure to outdoor smoke.

## Discussion

As climate change increasingly impacts health, environmental and social considerations are increasingly important in clinical, operational, and public health decisions. Existing efforts to integrate environmental and clinical data have succeeded in facilitating posthoc research to analyze the health impacts of social and climate variables and in developing large-scale public health surveillance and risk forecasting networks. The next step in developing climate-resilient health systems is the use of environmental data to inform interventions that mitigate these health impacts proactively [40].

Given regional variations in environmental exposures and in the population demographics that dictate vulnerability to these exposures, the utility of such systems will depend on their ability to process high-resolution and personalized data [36]. Even when environmental exposures are universally known to be harmful to human health, their specific health effects, and consequently the proactive interventions needed, will be variable according to the affected population. For example, a heat wave moving through a dense, urban area with an older population that has a high prevalence of cardiovascular disease may create a different risk profile than that of a younger, rural population with a high prevalence of asthma. Similarly, farm workers in Arizona may be at a higher risk of heat exhaustion than those in northern Maine, even with similar comorbidities, due to more extreme heat exposure. Existing information-rich data sources include national and regional temperature, water quality, and air quality data obtained from local or remote sensors. Further expansion of this infrastructure will be necessary to ensure adequate coverage, particularly for rural populations that may be most vulnerable to climate-related events.

Beyond the technical feasibility of performing environmental surveillance and integrating with medical data, logistical challenges include the dispersion of health data across multiple health care networks and electronic record systems, concerns regarding data privacy and security, and the cost of extracting and standardizing medical data [40]. A robust public health system to identify at-risk patients thus requires coordination among multiple relevant agencies, thoughtful approaches to managing data, and adequate funding for implementation. This coordination could happen at any level of governance, with existing efforts occurring both at nation- and city-wide scales. However, such coordination is expensive and may require reconceptualization of the financial incentives driving health care data management, which in their current state are likely obstructing data sharing and preventative care [36].

Environmental exposures related to climate change affect millions of people and cost billions of dollars annually, and a robust approach to connecting health and environmental records has the potential to improve both patient and system outcomes [1-4]. The requisite health and environmental data streams exist and continue to undergo improvement, but structural and financial barriers impede integration in a real-time manner that can support clinical, operational, and public health objectives [5,38,40]. By improving both reimbursement for such solutions and quantifying the utility of integrated environmental-health EHR systems, we will be better poised to address the impacts of environmental exposures on health and health care.

## Abbreviations

CMS: Centers for Medicare & Medicaid Services
COPD: Chronic Obstructive Pulmonary Disease
EHR: electronic health record
FHIR: Fast Healthcare Interoperability Resources
SDoH: Social determinants of health
Tracking Network: National Environmental Public Health Tracking Network
